# From Mitochondria to Atherosclerosis: The Inflammation Path

**DOI:** 10.3390/biomedicines9030258

**Published:** 2021-03-05

**Authors:** Juan M. Suárez-Rivero, Carmen J. Pastor-Maldonado, Suleva Povea-Cabello, Mónica Álvarez-Córdoba, Irene Villalón-García, Marta Talaverón-Rey, Alejandra Suárez-Carrillo, Manuel Munuera-Cabeza, José A. Sánchez-Alcázar

**Affiliations:** Andalusian Center for Developmental Biology (CABD-CSIC-Pablo de Olavide University) and Center for Biomedical Network Research on Rare Diseases, Carlos III Health Institute, 41013 Seville, Spain; juasuariv@gmail.com (J.M.S.-R.); carmenj3b@gmail.com (C.J.P.-M.); sulevapovea@gmail.com (S.P.-C.); monikalvarez11@hotmail.com (M.Á.-C.); villalon.irene@gmail.com (I.V.-G.); martatalrey@gmail.com (M.T.-R.); asuacar1@alu.upo.es (A.S.-C.); mmuncab@upo.es (M.M.-C.)

**Keywords:** atherosclerosis, mitochondria, inflammation, inflammasome, reactive oxygen species, NLRP3

## Abstract

Inflammation is a key process in metazoan organisms due to its relevance for innate defense against infections and tissue damage. However, inflammation is also implicated in pathological processes such as atherosclerosis. Atherosclerosis is a chronic inflammatory disease of the arterial wall where unstable atherosclerotic plaque rupture causing platelet aggregation and thrombosis may compromise the arterial lumen, leading to acute or chronic ischemic syndromes. In this review, we will focus on the role of mitochondria in atherosclerosis while keeping inflammation as a link. Mitochondria are the main source of cellular energy. Under stress, mitochondria are also capable of controlling inflammation through the production of reactive oxygen species (ROS) and the release of mitochondrial components, such as mitochondrial DNA (mtDNA), into the cytoplasm or into the extracellular matrix, where they act as danger signals when recognized by innate immune receptors. Primary or secondary mitochondrial dysfunctions are associated with the initiation and progression of atherosclerosis by elevating the production of ROS, altering mitochondrial dynamics and energy supply, as well as promoting inflammation. Knowing and understanding the pathways behind mitochondrial-based inflammation in atheroma progression is essential to discovering alternative or complementary treatments.

## 1. Mitochondria and Inflammation

Mitochondria are one of the most multifunctional organelles in the cell [1]. Their major function as cell energy generators has been extensively studied. In addition, mitochondria are involved in many cell processes, such as steroid biosynthesis [2], calcium [3] and iron [4] homeostasis, immune cell activation [5], redox signaling [6], apoptosis [7], and inflammation [8].

The first line of defense of metazoan organisms to deal with infection and/or tissue damage is the innate immunity response [9]. During infection, inflammation is commonly caused by microbial compounds, known as pathogen-associated molecule patterns (PAMPs), for instance, lipopolysaccharides (LPS) from bacteria or viral RNA. On the other hand, during tissue damage, inflammation is activated by intracellular molecules that are not usually exposed to the immune system. However, during cell stress or damage, they are secreted into the cytoplasm or leak into the extracellular environment. These molecules are called damage-associated molecular patterns (DAMPs).

Pattern recognition receptors (PRRs), including Toll-like receptors (TLRs), RIG-I-like receptors (RLRs), NOD-like receptors (NLRs), and C-type lectin receptors (CLRs), are sensors that recognize both PAMPs and DAMPs. The ligation of PRRs by DAMPs induces intracellular signaling pathways that promote the expression and activation of several pro-inflammatory mechanisms whose regulation and response depend on the PRR and cell type. Curiously, some PRR can ligate with both DAMPs and PAMPs [10]. The stimulation of a PRR through its ligands triggers the activation of the immune system [11]. For instance, DAMPs such as uric acid promote dendritic cell maturation [12] while PAMPs such as chitin, a major component of the fungal cell wall, can be recognized by epidermal cells for chemokine secretion and leukocyte recruitment [13]. Normally, the sensing of PAMPs or DAMPs by PRRs leads to the nuclear translocation of transcription factors, including the nuclear factor kappa-light-chain-enhancer of activated B cells (NF-κB), which upregulate the transcription of genes involved in the expression of pro-inflammatory cytokines, type I interferons (IFNs), chemokines and antimicrobial proteins, proteins involved in the modulation of PRR signaling, and inflammasome proteins [14].

The close similarity between prokaryotic DNA and mitochondrial DNA (mtDNA) is an important factor for understanding the role of mitochondria in inflammation, as well as supporting evidence for the endosymbiosis theory [15]. mtDNA is a double-stranded, circular molecule that contains a high concentration of cytosine-guanine sequences or CpG islands. mtDNA is released by damaged cells and can be sensed by a PRR, the Toll-like receptor 9 (TLR9), which is the receptor for CpG motifs in DNA [16]. This interaction leads to the NF-κB activation signaling pathway and, consequently, the induction of multiple pro-inflammatory genes [17,18]. In addition, mtDNA can activate the nod like receptor family pyrin domain containing 3 (NLRP3) inflammasome [19], thereafter promoting caspase-1 activation, the processing of interleukin 1β (IL-1β) and interleukin 18 (IL-18), and eventually, cell death. Mitochondrial dysfunction may also amplify the activation of NLRP3 by mitochondrial ROS production. It is well known that these oxygen species bind and activate NLRP3 in a continuous vicious cycle where NLRP3 will also promote ROS generation [20]. The stimulator of interferon genes (STING) inflammatory pathway can also be activated via mtDNA by the protein cyclic GMP-AMP synthase (cGAS) [21]. In this case, it will result in increased interferon-regulatory factor 3 (IRF3)-dependent gene expression, including induction of type I interferons. In summary, mtDNA is able to activate the innate immune signaling pathways by acting as a danger signal released from mitochondria into the cytoplasm, which warns the cell of serious damage.

Mitochondria are also necessary for the oligomerization of retinoic-acid-inducible gene I (RIG-I)-like receptors (RLR), which are involved in the recognition of the viral double-stranded RNA PAMP. After viral RNA is detected by RIG-I in the cytosol, it interacts with the mitochondrial antiviral signaling protein (MAVS), located in the outer mitochondrial membrane. Then MAVS is activated and recruits the machinery involved in type I interferon production [8,22]. MAVS also participates in other signal pathways related with mitochondrial reactive oxygen species (mtROS) and inflammation [23,24]. Mitochondrial activity is also necessary for the Toll-like receptor (TLR) signaling cascades: activated TLRs can signal through tumor-necrosis-factor-receptor-associated factor 6 (TRAF), which translocates to the mitochondrion and ubiquitinates the mitochondrial protein evolutionarily conserved signaling intermediate in Toll pathway (ECSIT). This causes the mitochondrion to both move to the phagosome and enhance mtROS production, resulting in direct antimicrobial killing [25,26]. For instance, TLR1, TLR2, and TLR4 recognize bacterial tri-acylated lipopeptides and LPS in order to enhance ROS production in the phagosome (oxidative burst of macrophages), which is required for its antimicrobial activity [27].

Although mitochondria are highly related to many inflammatory pathways, in this review, we will focus on NLRP3 activation, since it has proven to be a key event in the inflammatory process of atherosclerosis [28] and its relationship with mitochondria [29].

## 2. Atherosclerosis as a Representative Inflammatory Disease

Atherosclerosis is recognized as a chronic inflammatory disease characterized by the accumulation of lipids, mainly cholesterol, and other components, such as fatty substances, cellular waste products, calcium, and fibrin within the arterial wall [30]. Atherosclerosis is virtually the primary cause of cardiovascular disease (CVD)-related events, including myocardial infarction and stroke. CVD is the leading cause of mortality worldwide, accounting for almost 17 million deaths every year [31], approximately one-third of the total global deaths. The development of atherosclerosis is primordially thought to be the result of a previous dyslipidemia, mostly hyperlipidemia [32]. However, the precise initiating event of atherosclerosis seems to be multifactorial and remains unknown. Both high-density lipoprotein (HDL) and low-density lipoprotein (LDL) are essential for cholesterol transport and have been associated with atherosclerosis progression [33]. Specifically, elevated levels of LDL cholesterol (LDL-C) have been related to atherosclerosis progression [34]. LDLs retained in the extracellular matrix mainly by proteoglycans become targets for oxidative and enzymatic modifications. Then, oxidized LDLs (oxLDLs) lead to pro-inflammatory reactions, promoting the activation and recruitment of monocytes and other inflammatory cells trafficking across the vessel wall [35]. Mutations in proteins related with cholesterol production and transport, such as LDL receptor (LDL-R) and apolipoprotein B (Apo B), will usually result in familial hypercholesterolemia, a group of genetic disorders characterized by highly elevated plasma total-cholesterol levels and an early onset of atherosclerosis [36,37].

**Figure 1:**
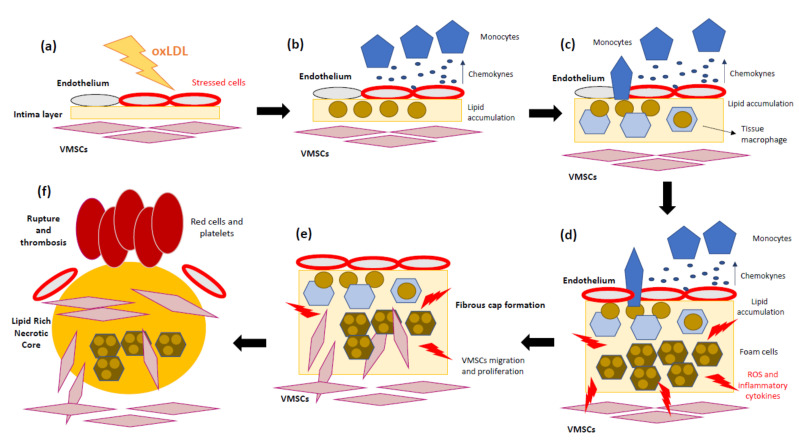
Schematic overview of atherosclerosis progression. (**a**) Oxidized low-density lipoprotein (oxLDL) contributes to the initial lesion in the arterial wall. (**b**) Endothelium cells produce pro-inflammatory cytokines, and circulating monocytes are recruited. (**c**) Monocytes migrate into the intima and differentiate into tissue macrophages. (**d**) Once in the artery wall, macrophages engulf the excessive lipids and become lipid-laden foam cells, which can accumulate and form a fatty streak. During the complex lesion progression, foam cell lysis by apoptosis and necrosis leads to the formation of a necrotic core and, together with defective efferocytosis, leads to the amplification of the inflammatory response. (**e**) Vascular smooth muscle cells (VSMCs) migrate from the media to the intima, where they differentiate into proliferative synthetic cells that generate extracellular matrix to form the fibrous cap and hence stabilize plaques. (**f**) During later stages, the plaque can become unstable due to the inhibition of extracellular matrix (ECM) formation, particularly collagen production by VSMCs. In addition, ECM is degraded by proteases released by macrophages, resulting in an unstable lesion that can rupture and lead to thrombosis.

Several studies support not only lipid accumulation but also the key role of the inflammatory mechanisms in atherosclerosis progression [38,39,40]. To sum up, the pathomechanisms of atherosclerosis involve the following steps: (1) Endothelial cells suffer inflammatory activation caused by oxLDL [41]. (2) Monocytes and other leukocytes are recruited in response to endothelium stress. (3) Chemokines and chemoattractant proteins alert inflammatory cells to migrate into the intima layer. (4) Monocytes differentiate into macrophages, which internalize LDL-C and transform into foam cells. (5) Foam cells and inflammatory macrophages release inflammatory cytokines and ROS. (6) Accumulation of dead foam cells will eventually form the lipid or necrotic core of the mature plaque. (7) The arrival of new immune cells amplifies the local inflammatory response and weakens the plaque’s fibrous cap. In addition, matrix metalloproteases (MMPs) promote vascular smooth muscle growth, while conversely provoking focal destruction of the vascular extracellular matrix, facilitating plaque rupture [42]. (8) Finally, the fracture or erosion of a weakened fibrous cap allows the exposition of thrombogenic components of the necrotic core of the plaque [43]. These steps are represented in Figure 1. As mentioned above, inflammation has a relevant role in the first steps of atherosclerotic lesions and promotes most of the thrombotic plaque complications [44].

Inflammatory responses can be triggered by the formation of the inflammasome, a cytoplasmic multimeric protein complex that is assembled in response to DAMPs or PAMPs [45]. The canonical inflammasome assembly starts with the activation of nod-like receptors (NLRs) or AIM2-like receptors (ALR) that induce the initial inflammatory signaling. The inflammasome holds the NLR and ALR sensors, adaptors such as Apoptosis-associated Speck-like protein containing a Caspase recruitment domain (ASC), and effectors such as caspase-1 [46]. Caspase-1 is considered an interleukin 1 (IL-1) converting enzyme (ICE) [47]. Through inflammasome activation, caspase-1 is able to convert pro-IL-1β into mature IL-1β, which is secreted to initiate inflammatory responses. Caspase-1 can also maturate pro-IL-18 into IL-18, which is related with IL-1 family cytokines [48]. Several distinct inflammasomes have been identified, among them some named after the NLR protein they contain, such as NLRP1, NLRP3, NLRC4, NLRP6, and NLRP12 [49]. Thus, for instance, NLRP3 inflammasomes are composed of NLRP3 protein, ASC, and caspase-1 [50]. Inflammasomes can be activated by diverse factors from different sources, such as extracellular adenosine triphosphate (ATP) or intracellular mtDNA. However, it is still unknown how NLRP3 can detect DAMPs [51]. There are very clear NLRP3 inflammasome activators, such as alterations in the potassium (K^+^) efflux, generation of mitochondrial ROS, and lysosomal destabilization [52]. Two main signals are necessary for NLRP3 inflammasome activation: The first one is called priming and requires signaling through pattern recognition receptors, such as TLRs, and the subsequent activation of NF-kB, which triggers the transcription of NLRP3 protein. The second one requires another activating signal, either mtDNA or ROS. Subsequently, NLRP3 assembles with ASC, and this complex recruits pro-caspase-1 for its maturation into its active form. Once the NLRP3 inflammasome is fully formed, pro-IL-β is processed into mature IL-1β, which is released by a mechanism independent of the Golgi apparatus [53,54].

As several experimental and clinical reports have demonstrated that IL-1β is a proatherogenic cytokine [55,56], NLRP3 inflammasome activation could contribute to the progression of atherosclerosis. Duewell et al. [57] demonstrated the relationship between NLRP3 inflammasomes and atherosclerosis. Using atherosclerosis-prone LDLR^−/−^ mice, they produced chimeric mice whose bone marrows were transplanted with NLRP3^−/−^, ASC^−/−^, or IL-1α/β^−/−^ bone marrow cells and showed that the lack of these inflammasome-related molecules significantly reduced the development of atherosclerotic lesions. Because cholesterol crystal formation is present in the early stages and can be detected in all stages of atherosclerosis, Duewell et al. focused on cholesterol crystals as a DAMP candidate and found that these crystals strongly activated NLRP3 inflammasomes in macrophages. Previously, it had been demonstrated that the presence of oxLDL can lead to cholesterol crystallization [58]. Therefore, the induction of NLRP3 and pro-IL-1β expression by oxLDL could promote IL-1β release. In addition, incorporation of oxLDL via a scavenger receptor CD36 provoked intracellular cholesterol crystallization in endothelial cells [59,60].

Nowadays, the NLRP3 inflammasome, a main generator of activated IL-1 family cytokines, is extensively studied due to its crucial role in the pathogenesis of atherosclerosis. Although NLRP3 inflammasome regulation is not clear, some mechanisms have been elucidated, such as its ubiquitination and phosphorylation, which induce post-transcriptional modifications that modulate its activation [61]. Until now, just one report has shown that hematopoietic NLRP3 deficiency in an LDLR^−/−^mouse model has small influences on atherogenesis progression [62]. In contrast, numerous studies have manifested the relevant significance of the NLRP3 inflammasome in atherosclerosis. For example, separate studies have verified that ApoE^−/−^ Caspase-1^−/−^ mice on an atherogenic diet show a significant reduction in atherosclerotic lesions [63,64]. In another study, NLRP3 downregulation in a diet-induced model of atherogenesis in double ApoE^−/−^mice prevented plaque progression and inhibited the maturation of pro-inflammatory cytokines [65].

Ultimately, NLRP3 activation in endothelial cells triggers the release of IL-1β [66]. Thus, increased levels of IL-1β were observed in atherosclerotic patients and were positively correlated to disease severity [67]. Furthermore, IL-1β acts on most cells present in the atheroma. In the endothelium, it induces procoagulant activity, increased expression of adhesion molecules for leukocyte recruitment, and production of monocyte chemoattractant protein 1 (MCP-1) [68]. All these changes allow the recruitment of monocytic phagocytes, which are strongly implicated in atherogenesis. IL-1β also acts on human vascular smooth muscle cells (VSMCs), a cell type that participates in all stages of atherosclerotic plaques, promoting their proliferation [69]. In this respect, it is worth mentioning the Canakinumab Anti-inflammatory Thrombosis Outcome Study (CANTOS) trial [60], a massive a randomized, double-blinded, placebo-controlled trial that investigated the use of canakinumab, a monoclonal antibody targeting IL-1β, on high-risk patients with established atherosclerotic disease who had already survived myocardial infarction. It was concluded that anti-inflammatory therapy targeting the IL-1β innate immunity pathway with canakinumab led to a significantly lower rate of recurrent cardiovascular events independent of lipid-level lowering, therefore bringing out the importance of inflammation in atherosclerosis [70].

There is a clear correlation between aortic NLRP3 expression and CVD prevalence [71]. It has been found that patients with coronary atherosclerosis display high aortic expression of NLRP3, which correlates with its severity [72]. Moreover, there is a significantly up-regulation of NLRP3 inflammasome components as well as mature IL-1β and IL-18 in human carotid atherosclerotic plaque tissue obtained by carotid endarterectomy in comparison to non-atherosclerotic mesenteric or iliac arteries [73,74]. NLRP3 expression levels were significantly higher in symptomatic compared with asymptomatic patients [74], and NLRP3 inflammasome components and signaling were highly expressed in unstable compared with stable atherosclerotic plaques [73]. For this reason, it could be interesting to consider NLRP3 protein expression levels in peripheral blood monocytes as a biomarker for predicting atherogenic complications in patients with CVD [75].

## 3. The Role of Mitochondria in Atherosclerosis

Mitochondria are the major source of energy in the cell through their mitochondrial respiratory chain (MRC). Almost all the energy released during mitochondrial electron transport is used for ATP synthesis, making MRC one of the most refined processes in nature. However, just a small percentage of electrons leak to oxygen, resulting in the generation of superoxide radicals, which are considered mitochondrial ROS [76]. There are alternative sources of hydrogen peroxide (H_2_O_2_) and superoxide radical anion (O_2_^−^), such as the mitochondrial outer membrane enzymes monoamine oxidase [77] and aldehyde oxidase [78].

ROS are necessary for the cell as secondary signaling agents [79], and they are regulated by numerous antioxidant molecules and proteins. In fact, ROS are gaining relevance in many research fields since it is now known that they can function as signaling molecules and in protein modification processes [80,81]. This mechanism is carried out by the nicotinamide adenine dinucleotide phosphate (NADPH) oxidase enzymes, whose sole function is the formation of ROS. Seven NADPH oxidases are expressed in the human body, namely Nox1–Nox5 and Duox1 and Duox2 [82]. Each one has different functions, for instance, Nox2 is involved in host defense [81], Nox4 plays a role in cellular homeostasis and cancer [83], and Nox5 is related with cardiovascular health [84].

In mitochondria, O_2_^-^ is rapidly transformed into H_2_O_2_ by manganese-dependent superoxide dismutase (SOD2), followed by its conversion into water by glutathione peroxidase 1 (GPX1) [85]. Under stress or pathological conditions, ROS are released from different sources, such as the activity of xanthine oxidase, lipoxygenase, nicotinamide adenine dinucleotide phosphate oxidase, uncoupling of nitric oxide (NO) synthase, and the leakage of electrons at mitochondrial complexes I and III during oxidative phosphorylation (OXPHOS) [86]. Therefore, damaged mitochondria produce large amounts of ROS, which in turn can affect the function of adjacent mitochondria. This continuous oxidative cycle is named ROS-induced ROS and is a common phenomenon based on the amplification of ROS production that induces further mitochondrial and cell dysfunction [87]. Interestingly, it has been reported that cytochrome c, a heme-containing protein mainly involved in mitochondrial electron transport, is involved in the ROS-induced ROS process [88].

Under normal physiological conditions, ROS damage is controlled by antioxidant molecules, such as glutathione, carotenoids, coenzyme Q_10_ (CoQ_10_), and antioxidant enzymes. However, in atherosclerosis, when ROS surpass the antioxidant barriers, the increased oxidative stress in the arterial endothelium triggers the appearance of oxLDL [89]. In addition, this process drives an ROS-induced ROS mechanism, increasing mitochondrial ROS and promoting atherosclerosis progression [90]. An interesting study has shown that the accumulation of ROS also promotes DNA fragmentation and increases monocytes’ apoptosis in normocholesterolemic old mice, which is worsened in age-matched atherosclerotic mice, indicating that increased ROS may promote the aggravation of age-related atherosclerosis [91]. In addition, the aggregation of LDL-C in the arterial wall induces ROS production and enhances atherosclerosis progression in hypercholesterolemic mice [92]. Moreover, mice with transplanted bone marrow with mitochondrial dysfunction showed increased ROS levels and apoptotic cells, which contributed to the development of pro-atherosclerotic aortic lesions [93,94].

There are several factors related with ROS production in atherosclerosis, including sex, age, exercise, diet, obesity, smoking, hypertension, diabetes, and hyperlipidemia. Although ROS could act as the second messenger in various cellular pathways, their accumulation can cause the activation of inflammatory cytokines [95]. Moreover, severe DNA damage caused by excessive ROS overactivates the poly(Adenosine diphosphate (ADP)-ribose) polymerase 1 (PARP1) pathway, which leads to the functional impairment or death of VSMCs and vascular endothelial cells (VECs) through ATP and nicotinamide adenine dinucleotide (NAD^+^) depletion and, consequently, increases inflammation. In addition, mitochondrial malfunction results in ROS overproduction, which induces the oxidation of lipids, nucleic acids, and proteins, which eventually leads to severe cellular damage. Enhanced ROS production causes endothelial dysfunction, vascular inflammation, and accumulation of oxLDL in the arterial wall, which are responsible for the formation of the early plaque and its growth [96]. Taken together, these findings indicate that mitochondrial dysfunction, in combination with oxLDL, originates a continuous cycle associated with inflammation that will eventually lead to atheroma formation.

Apart from ROS, mitochondria are also able to release mitochondrial-derived damage-associated molecular patterns (mito-DAMPs), which show immunogenic capacity when misplaced or imbalanced. Mito-DAMPs are known as early inflammatory modulators in response to cellular stress, promoting the chemoattraction of immune cells [97]. Although mito-DAMPs could have a positive effect on tissue injury recovery [98], aberrant and chronical mito-DAMP release could lead to severe mitochondrial dysfunction and continuous inflammatory processes, leading to pathological disorders [97,98,99].

One proposed mitochondrial mechanism to enhance cell survival and reduce atherosclerotic damage is the release of humanin, a prominent member of a newly discovered family of mitochondrial-derived peptides expressed from an open reading frame of mitochondrial 16S rRNA. This mitochondrial-encoded peptide has been shown to play a role in preventing cell death among various tissues [100,101,102], including the endothelium [103]. Zacharias et al. demonstrated that humanin is expressed in the endothelium, smooth muscles, and macrophages during atherosclerosis and that its administration ex vivo results in decreased ROS production and apoptosis after oxLDL exposure of human aortic endothelial cells [104]. They also showed that humanin exerts a protective effect on endothelial function and atherosclerotic progression in ApoE-deficient mice [105]. The specific signal leading to a higher expression of humanin in atherosclerosis is unknown. Since apoptosis is a natural process in late-stage atherosclerosis contributing to the formation of a necrotic core and unstable plaque, the expression of humanin might be a defense mechanism to slow down the progression of the disease. Although unstable plaques present higher humanin levels, this compensatory response may not be sufficient to withstand sustained damage and, consequently, eventual ischemic events might arise [104].

### 3.1. Focusing on the Endothelial Origin of Atherosclerosis

**Figure 2:**
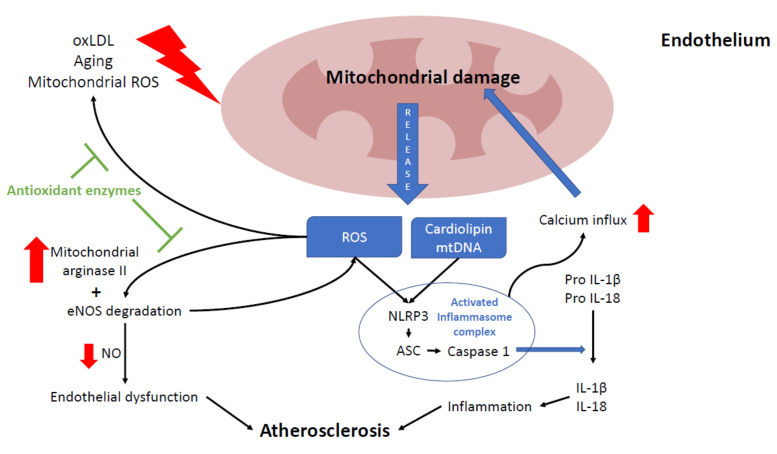
Relationship between mitochondria and atherosclerosis in the endothelium. Mitochondria play a key role in endothelium function, participating in several processes, such as nitric acid production, intracellular signaling, and cell death. Excessive reactive oxygen species (ROS) production leads to endothelium senescence, apoptosis, and atherosclerosis progression. Mitochondria can be damaged by oxLDL, ROS (mostly from mitochondrial origin), and the aging process. Damaged mitochondria release several mitochondrial components such as mtROS, cardiolipin and mtDNA which induce nod like receptor family pyrin domain containing 3 (NLRP3) inflammasome activation, leading to inflammation by increasing interleukin 1β and 18 maturation. Chronic NLRP3 activation will eventually lead to more mitochondrial damage by promoting mitochondrial calcium influx. Also, higher ROS levels disrupt the NO balance by boosting mitochondrial arginase II activity and causing eNOS degradation. In endothelium, the reduction of NO levels may also promote endothelial dysfunction and atherosclerosis. Most of these ROS-derived alterations are prevented by antioxidants enzymes (green block arrows).

Endothelial cells keep vascular homeostasis mostly by regulating vasodilation, platelet activation, leukocyte adhesion, and VSMC proliferation and migration [106]. Endothelial alteration is the first phase in early atherosclerosis (Figure 2), characterized by reduced NO secretion and synthesis [107]. NO prevents the expression of endothelium adhesion molecules and chemokines, as well as inhibits platelets’ activation and aggregation. These phenomena suggest that endothelial NO plays an important anti-inflammatory and anti-thrombotic role [108].

In the endothelium, NO is mostly synthesized by the conversion of L-arginine into L-citrulline by endothelial nitric oxide synthase (eNOS). NO levels are mainly regulated by intracellular arginine levels, which in turn are controlled by mitochondrial arginase II. Therefore, NO requires a healthy MRC function to maintain appropriate levels [109]. Reduced NO levels in the cell are mainly caused by eNOS degradation due to high levels of ROS and loss of mitochondrial membrane potential (ΔΨm) [107,108]. In addition, eNOS inhibition contributes to more ROS production, given that it is also necessary for mitochondrial biogenesis [110], thus damaging endothelial cells and promoting the development of atherosclerosis [111]. In mice, the overexpression of mitochondrial arginase II in endothelial cells reduces NO levels [112]. The loss of this equilibrium was also reported in the human endothelium with high levels of oxLDL, which cause an increase in arginase II activity [113].

The peroxisome proliferator-activated receptor γ (PPARγ) helper activator-1α (PPARγ coactivator-1α (PGC-1α)) is one of the most important regulators of mitochondrial biosynthesis in most cells, including endothelial cells [114], and it is considered a key protein in mitochondrial homeostasis. In addition, PGC-1α controls the activity of mitochondrial transcript factors A and B, which coordinate mtDNA expression [115]. PGC-1α expression inhibits the damage of endothelium-dependent vasodilation induced by fatty acids and restores the function of NO. Won et al. [116] revealed that PGC-1α restored endothelial cell fatty acid oxidation, optimizing the ATP/ADP translocator activity and increasing ATP synthesis. These effects allow the control of ROS levels, maintaining endothelial cells’ activity and stress resistance. In fact, the overexpression of PGC-1α promotes NO production and both independently inhibit ROS generation, thereby delaying atherosclerosis appearance. The most upstream pro-inflammatory effector in atherosclerosis is NF-kB [117], and the presence of PGC-1α can decrease the activity of NF-kB and tumor necrosis factor α (TNFα), consequently blocking oxLDL-related inflammation, regulating vascular endothelial growth factor-1 (VEGF1) expression, and stimulating angiogenesis [107]. In conclusion, the activity of PGC-1α prevents apoptosis, restricts inflammatory activation, and increases NO synthesis [115].

Recently, the mitochondrial production of peroxynitrite (ONOO^−^), a nitrogen reactive species (RNS), has been gaining relevance in cancer, innate immunity, vascular diseases, and inflammation [118]. In the endothelium, the presence of this RNS is regulated by thioredoxin reductase 2 (TrxR2) via the steady-state concentration of ONOO^−^, the reaction product of superoxide radical and nitric oxide, and the integrity of the vascular system [119]. Kameritsch et al. showed that mice with endothelial deletion of the *Trxrd2* gene develop increased vascular stiffness, hypertrophy of the vascular wall, and renal dysfunction [120]. In addition, increased ONOO^−^ production was detected in LPS-treated mice and this overproduction of ONOO^−^ was proportional to the developing progression of inflammation [121]. Impaired NO production was also related with enhanced ONOO^−^ production [122]. All of these factors taken together, a high concentration of ONOO^−^ has been directly linked with severe atherosclerosis damage [123].

### 3.2. Mitochondria and NLRP3 Inflammasome

Early evidence supporting the relationship between mitochondria and the NLRP3 inflammasome was reported by Zhou et al. [17], demonstrating that mitochondrial ROS are NLRP3 activators. Therefore, antioxidant compounds could block NLRP3 inflammasome assembly and ameliorate inflammation [124]. In addition, impaired mitophagy, a cellular process implicated in mitochondrial renewal, enhances mitochondrial damage and the release of ROS, mtDNA, and K^+^ into the cytoplasm, which promotes NLRP3 inflammasome activation. In fact, NRLP3 protein can interact directly with released mtDNA, initiating the inflammatory process [19]. Taken together, these findings support the hypothesis that impaired mitochondria could activate inflammation through NLRP3 in a direct way [125]. However, it is still unknown how NLRP3 activators can also induce mitochondrial damage. It is thought that they may alter intracellular Ca^2+^ homeostasis. One example is ATP, a canonical NLRP3 activator that can induce Ca^2+^ influx and promote the production of mitochondrial ROS and the loss of ΔΨm [126]. Furthermore, K^+^ efflux, caused by mitochondrial damage, can mediate the influx of Ca^2+^, resulting in a loss of mitochondrial Ca^2+^ homeostasis [127]. However, K^+^ efflux can also activate NLRP3 inflammasome independently of Ca^2+^ signaling [128]. New studies are required to evaluate the relevance of Ca^2+^ flux in NLRP3 inflammasome activation and mitochondrial dysfunction.

NLRP3 inflammasome can also be activated through the presence of bacterial molecules, such as N-acetylglucosamine, which are able to inhibit and dissociate the glycolytic enzyme hexokinase. Hexokinase is associated with the voltage-dependent anion channel (VDAC) at the mitochondrial outer membrane [129]. The VDAC regulates mitochondrial ROS production [130], can release large molecules (including mtDNA) into the cytosol [131], and is localized to cardiolipin-rich regions an NLRP3 activator [132]. The interaction of hexokinase with VDAC protects cells from mitochondrial ROS production [130] and inhibits the sustained opening of the mitochondrial permeability transition pore (MPTP) [131]. In addition, metabolic perturbations that inhibit hexokinase function, such as treatment with glucose-6-phosphate, 2-deoxyglucose, or citrate, all lead to inflammasome activation [133]. This evidence indicates the close relationship between metabolic alterations affecting hexokinase function and localization and inflammatory processes.

In contrast, several studies have reported another link between mitochondrial damage and NLRP3 activation. Thus, Yu et al. suggested that mitochondrial damage could be a side effect of inflammasome activation and, thereby, a consequence rather than a cause [134]. Additionally, the specific mitochondrial ROS activation mechanism in NLRP3 is still unknown. Despite the fact that ROS scavengers such as N-acetyl-lysine (NAC) reduce the transcription of NLRP3 and pro-IL-1β, they have no apparent effect on mitochondrial ROS [135]. Furthermore, ROS generation was not affected in NLRP3^−/−^ cells in response to LPS and ATP treatment, although NLRP3 deficiency was able to prevent mitochondrial depolarization [19]. Nevertheless, we are still far from establishing a clear connection between mitochondria and NLRP3.

Mitochondria themselves can also contribute to NLRP3 inflammasome formation by acting as an assembly platform. Specifically, MAVS and mitofusin 2 (Mfn2) have been proposed to recruit the NLRP3 protein to mitochondria in response to viral infection or non-mitochondrial NLRP3 activators. In its native form, most of the NLRP3 protein resides on the endoplasmic reticulum (ER). Upon stimulation with NLRP3 inducers, NLRP3 and ASC colocalize with mitochondria-associated ER membranes (MAMs) in the perinuclear space [136]. MAMs are essential in the initiation and regulation of the innate immune system, which includes inflammation [137], in addition to Ca^2+^ signaling [138]. Zhang et al. [136] described the following process: (1) After NLRP3 inflammasome activation, mitochondria cluster around the Golgi apparatus. (2) Diacylglycerol (DAG) starts to accumulate in Golgi and recruits protein kinase D (PKD). (3) PKD contributes to ASC oligomerization and NLRP3 phosphorylation and release from MAMs. (4) A new, fully mature NLRP3 inflammasome is released into the cytoplasm. To prove this process, PKD inactivation causes the retention of NLRP3 protein at MAMs adjacent to Golgi apparatus and reduces NLRP3 inflammasome assembly and activation. On the other hand, overexpression of PKD leads to NLRP3 inflammasome overactivation and IL-1β release without stimulation [136].

Mitochondria are a continuous and dynamic network that maintains an equilibrium between fusion, fission, repair, sequestration, degradation via mitophagy or mitophagy-independent mechanisms, and biogenesis [139,140]. Extracellular ATP, a well-known NLRP3 activator, binds to the P2X7 receptor, inducing K^+^ efflux, which could cause mitochondrial disruption [127]. In turn, mitochondrial damage could lead to the release of molecules (including mitochondrial ROS and mtDNA) that trigger NLRP3 inflammasome assembly [141]. As mitophagy allows the elimination of damaged mitochondria, a healthy rate of mitochondrial renewal will inhibit NLRP3 activation [18]. In contrast, mitophagy impairment by the depletion of autophagic proteins, such as microtubule-associated proteins 1A/1B light chain 3B II (LC3B-II) and Beclin-1, blocks the removal of malfunctioning mitochondria. Accumulation of damaged mitochondria promotes ROS generation and activation of the MPTP, which eventually leads to mtDNA release [19] and, as a consequence, NLRP3 inflammasome activation [20]. Furthermore, caspase-1 expression is highly decreased in LPS-primed murine phagocytes treated with ethidium bromide, which diminishes mtDNA copy number [19]. Likewise, NLRP3 inflammasome activation promotes cytosolic mtDNA release, which is abolished in LPS-primed NLRP3^−/−^ macrophages in response to pro-inflammatory signals [142]. 

Many NLRP3 activators, such as oxLDL, induce alterations in ΔΨm, which in turn leads to an overproduction of mitochondrial ROS [17] and activation of Ca^2+^ signaling [143]. Furthermore, the inhibition of Complex I by rotenone induces the loss of ΔΨm, increases ROS production, and enhances NLRP3-dependent IL-1β secretion [17]. In atherosclerosis, NLRP3 activation has been correlated with mitochondrial impairment by in vitro experiments showing the involvement of lectin-type oxidized LDL receptor 1 (LOX-1), a major receptor for oxLDL, in promoting mitochondrial damage [144]. However, there is no evidence of this phenomenon in vivo. A study has reported that fatty-acid-mediated mitochondrial uncoupling facilitates NLRP3-independent interleukin 1α (IL-1α) release in vitro, but not that of IL-1β, and atherosclerosis development in vivo [145]. These findings are in disagreement with other reports regarding the role of IL-1β release upon inflammasome activation in atherosclerosis [56].

All the proposed mechanisms for NLRP3 inflammasome activation, such as oxidative stress, mitochondrial dysfunction [146], ER stress [147], and lysosome rupture [148], have been observed in atherosclerosis. In addition, exogenous Ca^2+^ has been proposed as a new inflammasome activator due to its high concentration in atherosclerotic plaques, specifically around necrotic regions [149]. Finally, mitochondrial dysfunction has been demonstrated in dyslipidemia-related genetic disorders such as familial hypercholesterolemia [150,151]. 

### 3.3. Mitochondrial Mutations and Atherogenesis

The appearance of atherosclerosis in mitochondrial diseases may result from a primary pathological mechanism or as a consequence of a secondary mechanism associated with diabetes, arterial hypertension, or hyperlipidemia [152]. Most frequently, patients harboring mutations in mtDNA may develop primary mitochondrial atherosclerosis. Although the precise mechanism is not clear, it is believed that primary mitochondrial atherosclerosis results from increased oxidative stress, mitophagy alterations, energy deficiency, accumulation of toxic metabolites, or NLRP3 inflammasome activation. Thus, atherosclerosis in mitochondrial disorders may occur even in the absence of recognized atherosclerosis risk factors, suggesting that atherosclerosis can be a primary consequence of mitochondrial defect. However, there is still no direct evidence relating mitochondrial dysfunction to atherosclerosis progression [152].

Mitochondria could be directly linked to atherosclerosis in several ways. For this reason, mitochondrial dysfunction or excessive ROS caused by mitochondrial genetic mutations could promote atherogenesis [152]. In fact, it has been demonstrated that some mitochondrial mutations may lead to chronic inflammatory processes associated with NLRP3 activation [153], a key pathogenic factor in atherosclerosis development. This group of genetic alterations and the associated mitochondrial dysfunction may enhance the mitochondrial release of mtDNA and other mitochondrial DAMPs, initiating an inflammatory response through NLRP3 inflammasome activation [154].

Mitochondrial ROS have been suggested to be the origin of an increased mutation rate in mtDNA, one that is up to 15 times higher than that of nuclear DNA [155]. On the other hand, the presence of multiple copies of mtDNA in single cells explains the phenomenon of heteroplasmy, which refers to the variable proportion of wild-type and mutant mtDNA copies within the cell or the tissue [156]. This proportion between different mtDNA varies with aging and is dependent on the cell type and the tissue [157]. The assessment of the mtDNA heteroplasmic mutation load, as well as the mtDNA copy number, is a plausible way to explain the focality of atherosclerotic lesions [158,159]. Thus, cells possessing the level of heteroplasmy exceeding a certain threshold may exhibit mitochondrial dysfunction, whereas in the adjacent cells with a lower mutational load, the respiratory chain remains fully functional [160]. A similar mosaic pattern can be observed in atherosclerosis, where healthy areas of arterial wall coexist with regions bearing different types of atherosclerotic lesions [161,162].

Nowadays, several studies assume a connection between mtDNA mutations and atherosclerosis [163,164,165]. These studies were performed using leukocytes and arterial wall samples from atherosclerotic human patients. Most mutations were related to mitochondrial ribosomes, mitochondrial transfer RNA, and various mitochondrial-encoded respiratory complex subunits. It has been proposed that the presence of these mutations promotes mitochondrial dysfunction and therefore ROS production, which enhances the appearance of atherosclerotic plaques and increases the thickness of the intima and medial layers in carotid arteries [164]. Interestingly, Sazonova et al. discovered the presence of several mtDNA mutations associated with patients presenting with carotid atherosclerosis as well as two single-nucleotide substitutions that negatively correlate with atherosclerotic lesions [164]. It has been proposed that these mutations can be biomarkers for assessing predisposition to this disease.

Despite the available evidence, there is no established consensus on the importance of heteroplasmy in atherosclerosis [166]. The main hypothesis suggests that a primary defect of the respiratory chain or OXPHOS system may be associated with reduced energy production that can ultimately lead to the collapse of cell energy metabolism. Uncoupling of the electron transfer from ATP synthesis results in the excess generation of ROS, leading to widespread cellular injury and vascular damage [167]. In addition, ROS overproduction may enhance the mtDNA mutation rate and, as a consequence, further increase ROS production [168].

One of the mechanisms that enable cells to cope with heteroplasmy and mitochondrial dysfunction is mitophagy. Mitophagy surveils the mitochondrial population, removing unnecessary and/or impaired organelles. [169]. This process requires high energy and regulates NLRP3 inflammasome activation [18,170]. Therefore, the defective removal of damaged mitochondria leads to the hyperactivation of inflammatory signaling pathways and, subsequently, to chronic local or systemic inflammation [171,172], which may result in focal or generalized atherosclerosis [173]. In agreement with this assumption, in vitro inhibition of mitophagy in a primary culture of human monocyte-derived macrophages increased pro-inflammatory response in the form of up-regulation of the IL-1β gene [174].

However, it is not clear whether it is the increased mitochondrial ROS caused by atherosclerosis throughout lifetime that increases somatic mtDNA mutations or if it is previous maternally inherited mtDNA mutations that promote atherosclerosis [166]. Overall, the mechanism behind the role of heteroplasmic mtDNA variants in the development of atherosclerosis is still not completely understood.

## 4. Inflammation as a Therapeutic Target

Statins, the canonical 3-hydroxy-3-methylglutaryl-coenzyme A (HMG-CoA) reductase enzyme inhibitors, are the most widely prescribed lipid-lowering drugs in CVD. There is evidence to suggest that statins, in addition to cholesterol reduction, have numerous beneficial effects, such as endothelial function improvement, immunomodulation, and antioxidant activity [175]. In addition, it has been reported that long-term statin treatment inhibits cardiac hypertrophy and cardiomyocyte apoptosis, skin atrophy, and osteoarthritis as well as improves age-associated endothelial dysfunction by suppressing the senescent phenotype through the amelioration of inflammation and oxidative stress [176,177]. Although the lipid-lowering mechanism of statins is well known, the complex network of their multiple beneficial and adverse effects is not completely understood [178]. Statins are highly recommended to prevent CVD not only because of their lipid modifying and cholesterol lowering properties but also because of their anti-inflammatory effect [179,180,181]. Statins’ main effect is blocking the synthesis of cholesterol targeting HMG-CoA reductase, the rate-controlling enzyme of the mevalonate pathway [181]. In addition, statins exert their beneficial effects through immunomodulatory functions. With regard to atorvastatin, a frequently prescribed statin, it has been reported that it blocks the NF-κB-dependent expression of NLRP3, as well as IL-1β production in the human monocytic cell line (THP-1) [182]. 

Statins, although generally well tolerated, are also related with some known side effects, such as muscle complaints, hepatic damage, and non-insulin-dependent diabetes [183]. Their popular use has shed light on several adverse effects that can result in intolerance and discontinuation of the treatment. Mild muscle symptoms affect up to 10–20% of statin-treated patients [184]. The most accepted hypothesis to explain statin side effects is based on statin-induced inhibition of HMG-CoA reductase, which besides cholesterol biosynthesis also participates in several cellular metabolic pathways, such as the biosynthesis of dolichols; prenylated proteins; and CoQ_10_, an essential cofactor required for electron transport between mitochondrial respiratory complexes. The decreased concentrations of CoQ_10_ in blood and skeletal muscles in statin-treated patients [185] has led to the hypothesis that statins may interfere with the MRC, which is necessary for muscle function, repair, and growth. Corroborating some degree of mitochondrial dysfunction, lactate blood levels were found to be significantly higher in patients treated with statins compared to controls [186]. Some patients also showed elevated lipid stores, ragged red fibers, and impaired mitochondrial function [187]. These alterations generally stop after the withdrawal of the statin treatment. However, some studies have not corroborated the implication of statins in mitochondrial dysfunction [188]. Most side effects depend on dosage, treatment period, atherosclerosis progression, and genetic background.

To avoid the potential statin-related mitochondrial dysfunction, the effect of boosting the OXPHOS system by mitochondrial function enhancers, such as CoQ_10_, has been extensively examined. Thus, several studies have evaluated the impact of CoQ_10_ supplementation on statin-associated muscle symptoms (SAMS), with approximately half of the participants showing beneficial effects and the other half showing no effect at all [189,190,191,192]. These results may be due to the imprecise nature of defining the symptoms. Thus, SAMs are very difficult to diagnose and treat because physicians have no reliable biomarkers and tests to confirm the diagnosis [193]. Furthermore, statins’ side effects are difficult to prove since patients usually take other medications that also produce myopathy as a side effect [194]. Overall, CoQ_10_ supplementation reduces most inflammatory parameters, including NLRP3 activation by restoring/enhancing mitochondrial function [195,196]. In addition, it has been demonstrated that CoQ_10_ reduces macrophage and lipid accumulation and foam cell formation in atherosclerotic patients [197,198]. In general, CoQ_10_ has a remarkable safety profile that shows a low rate of adverse events. Although CoQ_10_ is easily administered orally, CoQ_10_ supplementation presents several challenges, mainly derived from its poor bioavailability [199,200]. Exogenous CoQ_10_ is taken up from the intestine into circulation, with a range between 2% and 4% of the total uptake due to its poor solubility. Depending on the dosage, formulation, and dosing interval, blood levels of CoQ_10_ can be variable [201]. Interestingly, MitoQ, a potent antioxidant and CoQ_10_ analog that specifically targets mitochondria, has been shown to prevent adiposity, hypercholesterolemia, and hypertriglyceridemia associated with metabolic syndrome in ApoE^−/−^ mice by reducing mtROS [202]. Moreover, MitoQ improves vascular mitochondrial function in older adults and animal models [203].

The enzyme 5′ adenosine-monophosphate-activated protein kinase (AMPK) is a key cellular energy sensor involved in multiple cellular mechanisms, including mitochondrial network health, which is essential to maintain metabolic homeostasis [204]. The activation of AMPK has a high number of potential anti-atherosclerotic effects, including the reduction of inflammatory cell adhesion to blood vessel endothelium, lipid accumulation, and proliferation of inflammatory cells caused by oxidized lipids, as well as the stimulation of cellular antioxidant defenses, the inhibition of macrophages differentiation from monocytes, and the enhancement of enzymes responsible for NO formation [205,206]. Metformin is an AMPK activator that was identified as an anti-inflammatory agent that decreases pro-IL-1β and ROS [207]. It has been suggested that metformin targets Complex I of the MRC to reduce mitochondrial ROS production [208]. Metformin can inhibit pro-inflammatory responses and cytokine-induced nuclear NF-κB activation via AMPK in vascular endothelial cells [209]. In addition, metformin inhibits inflammatory responses via the AMPK-phosphatase and tensin homologue (PTEN) pathway in VSMCs [210]. On the other hand, PPARs have been implicated in the molecular pathway of atherosclerosis progression [211]. It has been suggested that thiazolidinediones (TDZ) such as pioglitazone, a group PPARγ agonists, can activate AMPK and increase the expression of genes involved in adiponectin signaling, mitochondrial function, and fat oxidation [212,213]. Recently, TDZ have shown to stabilize the atherosclerotic plaque in ApoE^−/−^ mice through the modulation of T cell adaptive immunity and AMPK activation [214]. Although TDZ are currently used as glucose regulators in diabetic patients, they have also been associated with a lower risk of non-fatal myocardial infarction, non-fatal stroke, and cardiovascular death [215]. In addition, AMPK early activation could help VSMCs’ early energetic switch between oxidative phosphorylation and glycolysis to bypass mitochondrial damage and stabilize the atherosclerotic plaque [203].

Other therapeutic strategies that involve mitochondria are under discussion. For instance, the inhibition of succinate dehydrogenase, a key enzyme in the Krebs cycle, by dimethyl malonate (DMM) may also provide a therapeutic benefit by limiting ROS production and pro-inflammatory pathways, as well as boosting the anti-inflammatory response [216]. In addition, the sirtuin (SIRT) protein family is gaining relevance due to its capacity to regulate a variety of genes encoding proteins that control inflammation, NAD^+/^NADH balance, and endothelial cell function [217]. One of the most popular SIRT activators is resveratrol, which is a polyphenol found in natural products, including grapes [218]. Resveratrol and its derivatives are strong activators of SIRT, with potentially anti-atherosclerotic effects [219,220].

Although there are only a few effective direct NLRP3 inhibitors, recently new results have been shown. MCC950, a diarylsulfonylurea-containing compound, was found to prevent the formation of the NLRP3 inflammasome by inhibiting the NLRP3-ASC oligomerization [221]. MCC950 was able to reduce LPS and nigericin-induced IL-1β [221] and the myocardial infarct size [222]. In atherosclerotic ApoE^−/−^ mice, MCC950 reduced atherosclerotic lesion development, which mainly resulted from a reduction in the number of macrophages inside the atherosclerotic plaque [223]. A direct NLRP3 inflammasome assembly inhibitor called CY-09 reduced inflammasome activation and shows remarkable therapeutic effects on mouse models of cryopyrin-associated autoinflammatory syndrome (CAPS) and type 2 diabetes [224]. Nowadays, new approaches using natural compounds inhibiting NLRP3 inflammasome are also relevant [225], for example, artemisinin [226], rosmaniric acid [227], curcumin [228], and berberine [229]. In addition, the proprotein convertase subtilisin/kexin type 9 (PCSK9), a well-known protein for its role in the LDL-R homeostasis and atherosclerosis progression, was shown to have a pro-inflammatory effect in macrophages [230]. PCSK9 inhibitors such as PCSK9 antibody therapy [231] would have a pleiotropic effect by reducing LDL-C and inflammatory response [232].

## 5. Conclusions

Mitochondria are critical regulators of many cell processes from energy production to cell death. Emerging knowledge about this organelle has shed light on its implication in inflammatory diseases. Nowadays, atherosclerosis is considered an inflammatory disease in which mitochondrial dysfunction may play an essential role. The most studied inflammatory process in atherosclerosis is related with NLRP3 inflammasome. In the endothelium, inflammation initiates a complex process that implies the participation of the immune system, VECs, and VSMCs, resulting in the formation, growth, and rupture of a lipid-rich necrotic plaque, with fatal consequences in vascular health. NLRP3 can be activated by several factors, including anormal potassium flux or the release of mitochondrial components, such as mtROS, cardiolipin, and mtDNA. Through this review, we discussed the close relationship between mitochondria and NLRP3 inflammasome and how primary or secondary mitochondrial damage can contribute to the initiation of the inflammatory response. The important role of mitochondria in atherosclerosis could explain partially why atherosclerosis is more prevalent on elderly people, due to the aging-related mitochondrial deterioration of endothelial and vascular smooth muscle cells. Thus, maintaining a healthy mitochondrial network is important for endothelial health. Although atherosclerosis can be prevented in hypercholesterolemic patients by statins, there are still serious side effects. New therapeutic targets focusing on mitochondrial network healthiness, such as antioxidants and direct inhibition of NLRP3, are promising therapeutic approaches in atherosclerosis. Understanding the biology and regulation of inflammasome-mitochondria connections is required to define the value of these mechanisms as potential therapeutic targets in atherosclerosis.

## Data Availability

Not applicable.

## Abbreviations

ADP	Adenosine diphosphate
AMP	Adenosine monophosphate
AMPK	5′ Adenosine-monophosphate-activated protein kinase
ApoE	Apolipoprotein E
ASC	Apoptosis-associated speck-like protein containing a caspase recruitment domain
ATP	Adenosine triphosphate
BRCA	Breast cancer 1
BRCC3	BRCA1/BRCA2-containing complex subunit 3
CD	Cluster of differentiation
cGAS	Cyclic GMP-AMP synthase
CoQ	Coenzyme Q_10_
CpG	Cytosine-guanine sequences
CVD	Cardiovascular disease
DAG	Diacylglycerol
DAMP	Damage-associated molecular pattern
DMM	Dimethyl malonate
eNOS	Endothelial nitric oxide synthase
ECM	Extracellular matrix
ECSIT	Evolutionarily conserved signaling intermediate in Toll pathway
ER	Endoplasmic reticulum
GMP	Guanosine monophosphate
GPX	Glutathione peroxidase
HDL	High-density lipoprotein
HMG-CoA	3-hydroxy-3-methylglutaryl-coenzyme A
ICE	IL converting enzyme
IL	Interleukin
IRF	Interferon-regulatory factor
LC3	Microtubule-associated protein 1A/1B-light chain 3
LDL-C	Low-density lipoprotein cholesterol
LDL-R	Low-density lipoprotein receptor
LOX	Lectin-type oxidized LDL receptor
LPS	Lipopolysaccharides
MAM	Mitochondria-associated ER membrane
MAVS	Mitochondrial antiviral signaling protein
MCP	Monocyte chemoattractant protein
Mfn	Mitofusin
MPT	Mitochondrial permeability transition pore
MRC	Mitochondrial respiratory chain
mtDNA	Mitochondrial deoxyribonucleic acid
NAC	N-acetyl-lysine
NAD	Nicotinamide adenine dinucleotide
NF-κβ	Nuclear factor kappa-light-chain-enhancer of activated B cells
NLR	Nod-like receptor
NLRP3	NLR family pyrin domain containing 3
NO	Nitric oxide
oxLDL	Oxidized low-density lipoprotein
OXPHOS	Oxidative phosphorylation
PAMP	Pathogen-associated molecule pattern
PARP	Poly(ADP-ribose) polymerase
PGC	PPAR helper activator
PKD	Protein kinase D
PPAR	Peroxisome-proliferator-activated receptor
PRR	Pattern recognition receptor
PCSK9	Proprotein convertase subtilisin/kexin type 9
PTEN	Phosphatase tensin homologue
RIG-I	Retinoic acid-inducible gene I
RLR	RIG-like receptor
RNS	Reactive nitrogen species
ROS	Reactive oxygen species
SAMS	Statin-associated muscle symptoms
SIRT	Sirtuin
SOD	Superoxide dismutase
STING	Stimulator of interferon genes
TDZ	Thiazolidinedione
TLR	Toll-like receptor
TRAF6	TNF-receptor-associated factor 6
VEC	Vascular endothelial cell
VSMC	Vascular smooth muscle cell
ΔΨm	Mitochondrial membrane potential
